# How French culture influences the framing of genetic modifications on the internet: Insights from Google-based corpus analysis

**DOI:** 10.1371/journal.pone.0311934

**Published:** 2024-12-23

**Authors:** Anaïs Degache, Séverine Louvel, Stéphanie Abrial, Virginie Tournay

**Affiliations:** 1 Aix Marseille University, IRD, LPED, Marseille, France; 2 Université Grenoble Alpes, CNRS, Sciences Po Grenoble, Pacte, Grenoble, France; 3 CNRS/CEVIPOF, SciencesPo, Paris, France; University of Malaya Faculty of Education, MALAYSIA

## Abstract

Since the mid-1990s, genetically modified (GM) crops and foodstuffs have been the subject of much controversy. While research has highlighted a disparity between attitudes toward the consumption of GM products, this study focuses on the circulation of cultural frameworks for GMs online. We use two datasets obtained using Google as a privileged observation site for understanding how debates regarding genetic engineering are framed in global and local contexts. While the English-language corpus brings to the fore the framing of GM products in terms of economic value, the French-language corpus is characterized by the strong association of such products with matters relating to risk. This has consequences for public perceptions of biotechnologies. Stakeholders using communication media to convey GM issues could assist public understanding by taking these cultural differences into account.

## Introduction

Since the 1990s, applications of genetic engineering in agriculture have multiplied. Faster and more precise tools—such as CRISPRCas-9—have produced new genetic variants which, from a molecular biology perspective, contain mutations that are indistinguishable from spontaneous mutations [1]. Nevertheless, the use of such emerging technologies in medicine and, markedly, in food production is a contentious subject, particularly within the European Union [2]. Public perceptions and understandings of these biotechnologies have serious consequences in the medical and agricultural fields, especially for regulation. Public attitudes toward biotechnologies vary considerably between European countries [3], and between Europe and the United States [4,5]. People’s acceptation of genetically modified organisms (GMOs) can be determined by personal values or worldviews [6], as well as by information about the type of genetic modification applied, and the labeling of genetically modified (GM). In particular, the lack of mandatory labeling is a significant contributor to the fact that US consumers are more accepting of GM foods than European consumers [7]. Consumer attitudes in Europe differ on the basis of whether the genetic modification is applied to an animal or a plant, uses genes from the same species or a different species, and whether the product has a direct tangible benefit to consumers, for example in improving nutrition. Lastly, medical applications such as prevention or cure of diseases, prevention of disabilities, and organ transplantation are much more desired purposes than improved plant production, livestock production, or changing non-life limiting characteristics of human embryos [3].

Information about new science circulating in the public domain is filtered by the media [8], which may affect how biotechnologies are perceived [9,10]. Numerous studies have compared how “new food technologies” have been covered by the media in different countries, over time, or in different types of media (the ‘elite press’, tabloids, political papers, the internet, etc.) [11–14], and have shown how GMOs are framed by the media. A frame is a narrative structure [15]: in this context emphasis may, for example, be placed on environmental risks, health risks, or on the socio-political implications of biotechnologies. Thus, “through frames, media highlight certain points of view and marginalize others, defining occurrences, and explaining how they are to be understood” [16: 184]. Among the variety of factors that influence media frames of biotechnology, national differences have been prominently investigated, such as country-based perceptions of social risk conveyed by GM foods [17]. Most studies of how media have covered “new food technologies” focus on elite newspapers, which are considered to be “opinion leaders”, and compare different countries with one another [18]. The importance of national and European legal frameworks has also been highlighted. For instance, it is now well established that in Europe, the legal debates on GMOs focused from the outset on the process, i.e., the genetic engineering or gene editing techniques for obtaining a GM product, and regulation complies with the precautionary principle (the purpose of which is to deal with uncertain risks likely to lead to serious and irreversible damage). By contrast, in the United States (US), the legal approach to GMOs focuses on the end products [19] and is based on the scientific evaluation of the benefit/risk balance of marketed products rather than whether or not they were obtained using genetic engineering techniques. How GMOs are portrayed in Europe’s media is influenced by the legal stance adopted by European legal institutions, which are more critical of GM products than North American ones [4].

In this paper, we attempt to understand the framing of agricultural biotechnology news on the internet, in a recent period (2018–2020) that was also an intense moment of controversy about how genetic engineering is used. In 2018, the Chinese scientist He Jiankui announced that he had edited the genomes of human embryos to confer resistance to HIV, leading to the birth of the twins “Lulu and Nana” on November 26, 2018. The same year, the Court of Justice of the European Union ruled that organisms engineered using New Plant Breeding Techniques (NPBTs) must comply with regulation on GMOs [20,21]. The choice of the period 2018–2020 is not intended to highlight the influence of predominant legal cultures in perceptions of risk assessments about organisms referred to as GM, but rather is intended to offer insights into the media framing of these biotechnologies in an intense moment of worldwide controversies.

The fact that the internet and social media have become significantly more influential in forming public opinion is recent. As the internet offers a public space for politically oriented conversation [22], we see it as a genuine echo chamber and a privileged site for observing the broader circulation of representations of topics relating to "genetically modified". GMO-related debates on the Internet are framed in different ways, and we wish to compare these frames according to whether the circulation of information is global or more localized. The internet is increasingly considered to be a legitimate information source [23,24] and is dominated by large and globalized corporations such as Google—the leading search engine accessible to all audiences—whose algorithms filter and select information conveyed on news websites. We chose to base our corpus of data on Google search results, in particular due to its preponderance in users’ choice of search engines and the algorithm’s ability to influence user decisions. It should also be emphasized that in this research, we exclude databases that are not fully accessible to standard search engines as in the “deep web”. According to Prasad [25], this part of the informational flow, that is not indexed by conventional search engines, accounts for more than 80% of internet traffic. Therefore, our focus is on a small share of online activity, but one that is generally accessible to all audiences without barriers to entry.

We hypothesize that the ways GMOs are framed differ in a globalized context vs. in specific national contexts. To test this hypothesis, we chose to analyze how two different languages—French and English—convey cultural specificities in the context of GMO-related communication. This choice was made to highlight the differences between globalized and localized information, rather than to focus on countries, as information circulates within, across and between countries. Here we consider English as a global language used both within and beyond English-speaking countries. English as a "global language" should be understood as Crystal [26] defines it: "a language achieves a genuinely global status when it develops a special role that is recognized in every country" [26: 3]. English is global because it is the first official language of several countries, where it can be used by all speakers as a native language, but also as an institutional language used by various organisations (such as European institutions, the US Food and Drug Administration, the English-language media, and multinational corporations) [27]. Moreover, English is global because even when it is not an official language, it is the first foreign language taught in schools. As so, when this "global English” is spoken, the conditions are not the same as when English is spoken by native speakers. Different expectations, practices, and norms prevail and apply [28]. By contrast, French is an international language, as it is spoken in several countries and is the official language of 29 countries. Yet it cannot be considered a global language because it does not achieve the economic and cultural functions of the English language, which are closely tied to the development of a global market and its dissemination in the fields of science, technology, culture, and media [29]. Therefore, although English may also refer to national contexts, it has a special role at the global level that French does not have.

In this paper, we address the ways in which way languages frame scientific controversies on the Internet, in the field of biotechnology that involves genetic modifications. We compare lexicons used in a global language (English) and in a language more closely tied to a national political culture (French) to reveal divergences in the understanding of genetic engineering, particularly with regard to how legal frameworks, science and production relate to one another. We argue that French-language internet content, although partially influenced by the worldwide circulation of English-language content, displays significant cultural variations with respect to GMOs compared to global English-language content.

## Methods

To answer our research questions, we have compiled an original corpus and analysed it with the tools of lexical statistics. We proceeded in three steps: first, we constituted two sets of documents: one in French, and the other in English. Second, we cleaned up the database, and third, we statistically analyzed the lexicon of texts.

### Procedure

We began by using the following Google query: “GMO” OR “GMOs” OR “NPBT” OR “NPBTs” OR “New Plant Breeding Techniques” OR “Genetically Modified Organism” OR “Genetically Modified Organisms” on Google.com. We searched by language: the English-language query only provided English-language pages, while the French-language query only provided results in French. The queries covered the period from January 2018 to July 2020 when the texts were grouped. Our aim in choosing this time period was to capture and understand the current communication relating to New Plant Breeding Techniques, starting after the Court of Justice of the European Union (CJEU) 2018 ruling that all New Plant Breeding Techniques should fall within the scope of EU legislation on genetically modified organisms (GMOs) [20]. The CJEU decision was a way to bring the legal framework of NPBTs in line with that inherited from GMOs, and we wanted to grasp the specificity of this legal ruling in terms of cultural framing of NPBT products.

First, we decided to include in our study all the texts and sites that mentioned GMOs or NPBTs at least once, even if they were advertising texts. Any webpage that did not meet these inclusion criteria was excluded from the corpus. We also excluded links to PDFs longer than two pages, scientific publications, or videos. A webpage that met the inclusion criterion but also had one or more exclusion criteria was excluded from the corpus. The two corpora were compiled by default by selecting the first web page linked by the Google search result. We selected only the textual content that provided information about the webpage, excluding menus, search bars, and any non-readable content. We normalized the content by removing accents and special characters so that the software could read the text. In this way, the content of each web page was collected into a single.txt file, one for each language.

Second, all texts were categorized to identify their characteristics:

- the **language**: French or English.
- the **year** of publication or last revision: 2018; 2019; 2020 as provided by Google.
- the **origin** of the text, chosen based on the declaration in the “About” section of each website: international or European if specified; if the origin is not specified or is too vague, or if the company, the magazine, or the association has at least one branch or edition in another country, the classification is “international” (for example NGOs such as Greenpeace, or corporations such as Syngenta). In other cases, the classification is “national”–and the country of origin is specified for the French corpus, or “unknown” if the origin cannot be traced.
- the main **issue** the text focuses on: “regulation”–websites and articles about the regulation of GMOs, NPBTs, or administrative sites; “bioagri”–websites and articles about agriculture, biodiversity, pesticides, and animals; “geopoeco”–geopolitics, economics, and patentability; “science”–for innovation, scientific popularization, risk assessment; and “marketing”–for all advertising websites.
- the main **stakeholder** depends on the **medium** chosen: for general websites, we traced the stakeholder by looking for the company or the organization hosting the text; however, for press articles and blogs, we looked for the description of the author (e.g. scientist, journalist, farmer), except for one-person interviews. The “citizen” categorization was chosen when the author is anonymous (for example in free encyclopedias) or cannot be placed in any other category.
- the **target** of the text: if the website, the magazine, or the blog is specialized in a particular field and specifically targets this sector, we classify it as “experts”. In all other cases, the target is “general public”.

We used the software Iramuteq (R Interface for Multidimensional Analysis of Texts and Questionnaires) to analyze our data. The software provides lemmatization of forms through an integrated dictionary. The lemmatization process involves grouping nouns, acronyms, verbs, and adjectives under their base forms while excluding prepositions, conjunctions, pronouns, and auxiliaries from the main analysis. We adapted the software dictionary to recognize related word forms in both English and French. For example, hyphenated forms such as "non-GMO," "well-being," and "gluten-free" were originally recognized as separate words. We identified and reassociated these forms within the software to treat them as a single entity. In addition, we differentiated forms containing "genetic" to prevent the software from aggregating them all under "genetic." Forms such as "genetically modified," "genetic modification," "genetic engineering," and "genetically engineered" were reassociated accordingly. This adjustment ensured that the software accurately processed and analyzed the specific lexical forms relevant to our study, thus improving the accuracy and relevance of our textual analysis across the English and French datasets.

### Materials

The English-language corpus contains 214 texts: 236,105 occurrences, 10,916 forms and 4,340 hapax (1.84% of occurrences, 39.76% of forms). The French-language corpus contains 146 texts: 128,547 occurrences, 8,666 forms, and 3,828 hapax (2.98% of occurrences, 44.17% of forms). Number of occurrences refers to the total number of words in the corpus. Forms are the words after a procedure of lemmatization. Hapax are words found only once in the corpus.

For the English-language corpus, the objective was to identify what can be found in a globalized language. 37.38% of the texts come from international non-European websites, 5.61% come from “Europeanunion” websites, and 52.34% from national websites. See Table 1 for the distribution of categories in the English-language corpus.

**Table 1:** Distribution of categories in the English-language corpus.

Code	Category	No. of texts	In %
**Year**	2018	68	31.78%
	2019	97	45.33%
	2020	49	22.90%
	TOTAL	**214**	100.00%
**Origin**	international	80	37.38%
	national	112	52.34%
	Europeanunion	12	5.61%
	unknown	10	4.67%
	TOTAL	**214**	100.00%
**Issue**	regulation	49	22.90%
	bioagri	23	10.75%
	geopoeco	23	10.75%
	science	82	38.32%
	marketing	37	17.29%
	TOTAL	**214**	100.00%
**stakeholder**	scientist	25	11.68%
	company	66	30.84%
	organization	59	27.57%
	journalist	54	25.23%
	farmer	5	2.34%
	citizen	5	2.34%
	TOTAL	**214**	100.00%
**medium**	press	63	29.44%
	encyclopedia	3	1.40%
	blog	17	7.94%
	Website	131	61.21%
	TOTAL	**214**	100.00%
**target**	generalpublic	158	73.83%
	experts	56	26.17%
	TOTAL	**214**	100.00%

For the French-language corpus and in contrast to the English-language corpus, the objective was to gain an insight into the specificity of a language embedded in stronger geographical and cultural contexts. A large majority of texts come from France (71.23%), 12.33% from international websites, and only 3.42% from non-French European websites. Other countries such as Canada (Québec), Switzerland, Belgium, and Lebanon contributed slightly to the corpus. See Table 2 for the distribution of categories in the French-language corpus.

**Table 2:** Distribution of categories in the French-language corpus.

Code	Category	No. de texts	In %
**Year**	2018	49	33.56%
	2019	51	34.93%
	2020	46	31.51%
	TOTAL	**146**	100.00%
**Origin**	International	18	12.33%
	Europeanunion	5	3.42%
	nationalFrance	104	71.23%
	nationalCanada	8	5.48%
	nationalSwitzerland	4	2.74%
	nationalBelgium	2	1.37%
	nationalLebanon	1	0.68%
	Unknown	4	2.74%
	TOTAL	**146**	100.00%
**Issue**	Regulation	39	26.71%
	Bioagri	31	21.23%
	Geopoeco	18	12.33%
	Science	33	22.60%
	Marketing	25	17.12%
	TOTAL	**146**	100.00%
**stakeholder**	Scientist	10	6.85%
	Company	28	19.18%
	organization	32	21.92%
	Journalist	64	43.84%
	Farmer	6	4.11%
	Citizen	6	4.11%
	TOTAL	**146**	100.00%
**medium**	Press	75	51.37%
	Encyclopedia	2	1.37%
	Blog	4	2.74%
	Website	65	44.52%
	TOTAL	**146**	100.00%
**target**	Generalpublic	131	89.73%
	Experts	15	10.27%
	TOTAL	**146**	100.00%

### Statistical analysis

Computer-assisted analysis of textual data involves multidimensional statistical analysis of texts, with frequency and specificity measurements, factor analysis of correspondence and automatic classification [30]. Iramuteq is based on the Descending Hierarchical Classification method introduced by Max Reinert [31]. The aim of this classification is to create groups of forms to see how these groups are connected to each other, through cluster hierarchical classification, as specified in Table 3. To initiate this descending hierarchical classification, the software begins by creating a large table in which each form is listed in rows and each "text segment" is listed in columns. A "text segment" is constructed based on the best ratio of size to punctuation. The software then classifies the text segments into clusters based on the occurrence of the forms in those text segments. The clustering calculation is based on the chi-squared (χ^2^) test with 1 degree of freedom, which helps determine the statistical significance of the observed occurrences in the text segments. The critical value for the chi-squared test at the 0.05 significance level is consistently 3.841.

**Table 3:** Iramuteq classification of groups in the English-language corpus and in the French-language corpus.

	English-language corpus	French-language corpus
Number of texts	214	146
Number of text segments	6519	3559
Number of forms	14366	12624
Number of occurrences	236105	128547
Number of lemmas	10916	8666
Number of active forms	9758	8028
Number of supplementary forms	1158	638
Number of clusters	5	5
% of segments classified	86.33%	91.06%

We were able to specify a maximum number of clusters to be created by the software. By creating 5 clusters, the software allowed us to classify 86.33% of the text segments in the English corpus and 91.06% in the French corpus. This was the best ratio between the number of clusters created and the number of text segments classified (see Table 3).

## Results

### Producing, regulating and consuming: The dominant approach to GMOs in the English-speaking world

**Fig 1:**
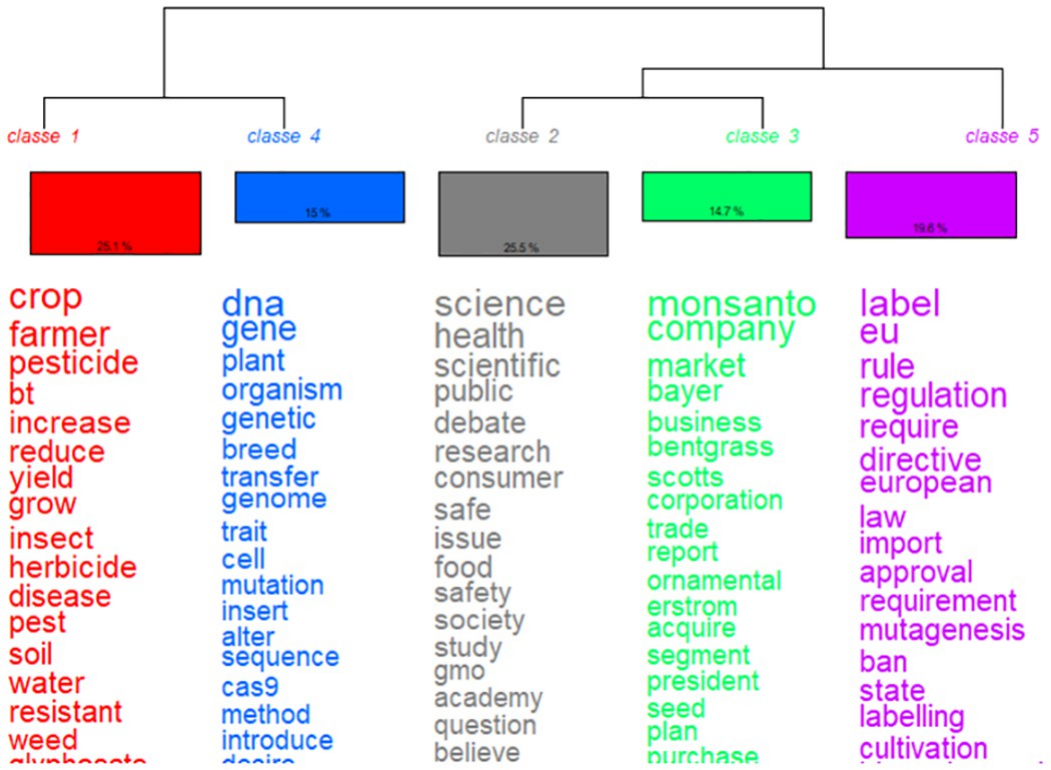
Descending Hierarchical Classification of the English corpus.

**Fig 2:**
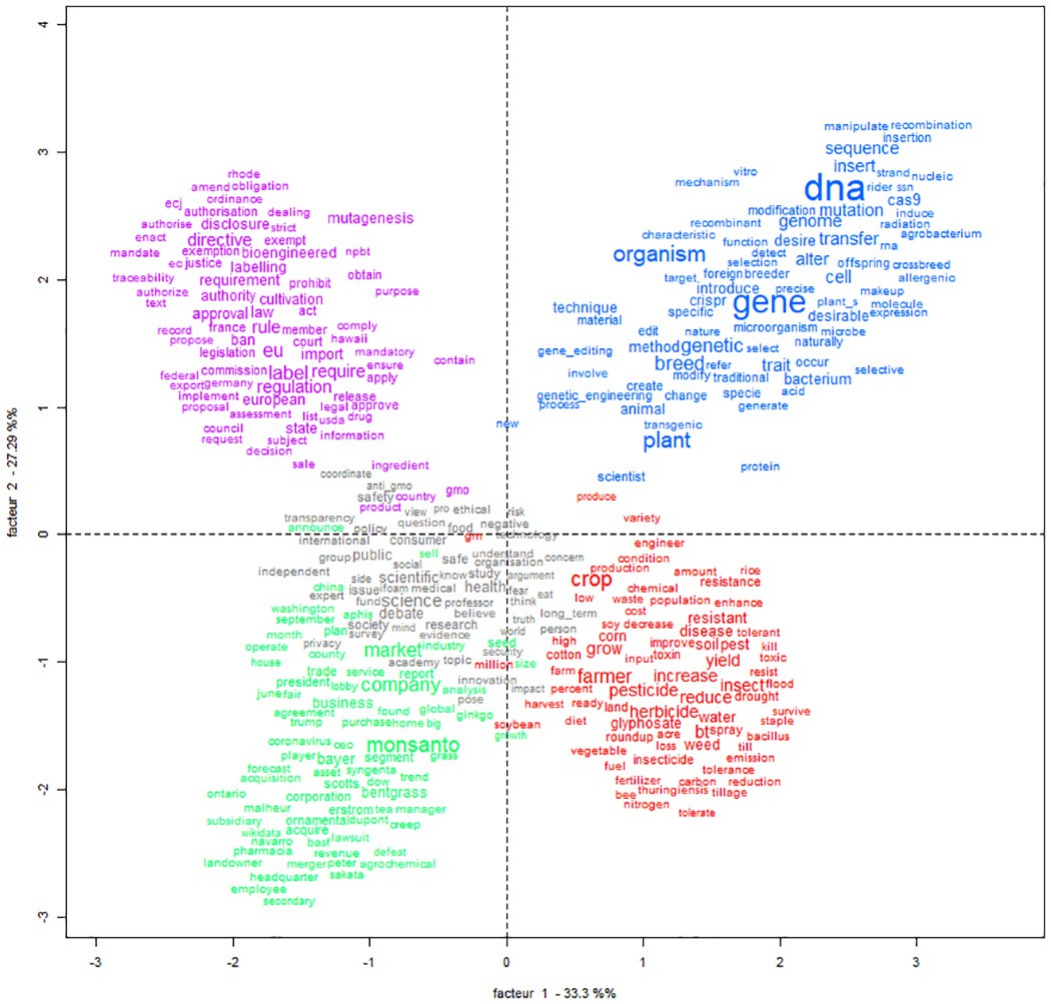
Correspondence factor analysis (CFA) of classes produced by Descending Hierarchical Classification (DCA) of the English corpus. Font size proportional to frequency by class. Horizontal axis, factor 1, 33.33%—Vertical axis, factor 2, 27.29%.

In the English-language corpus, the term “genetically modified” is above all used to refer to GM products that are standardized, regulated and marketed, but also cultivated and engineered (frequences seen in Table 4). The Reinert classification enables the two major contrasting lines structuring the lexical universes of the English-language corpus to be identified (cf. Fig 1, which shows the hierarchical classification and Fig 2, which projects the results of the hierarchical word classification of the English-language corpus on a factorial plane).

**Table 4:** The 30 most frequent words in the English corpus.

Form	Freq.	POS
gmo	3323	Nr
food	2247	Nom
crop	1600	Nom
plant	1155	Nom
product	814	Nom
new	692	Adj
gm	682	Nr
farmer	603	nom
seed	595	nom
gene	579	nom
organic	529	nom
label	502	ver
health	497	nom
grow	486	ver
include	467	ver
corn	463	nom
market	458	ver
year	449	nom
genetically_modified	448	Nr
company	421	nom
research	414	nom
technology	406	nom
consumer	405	nom
produce	397	nom
non_gmo	385	Nr
organism	371	nom
world	364	nom
genetic_engineering	359	Nr
breed	356	ver
ingredient	351	nom

The first opposition is between institutional positioning—with three sets of discourse: on “labeling” (in magenta), “research governance” (in gray), and “commercialization” (in green)—and the professional use of genetic engineering techniques in laboratories or agriculture—illustrated by discourses on “biosciences” (in blue) and “agricultural practices” (in red). The total variance of the correspondence analysis is 91.02%. This value represents the sum of the variability explained by all the classes in our contingency table. This shows that the identified classes capture almost all of the observed variance in the data. More specifically, the observed variance for each class shows that all classes contribute to the structuring of the data. For "agricultural practices," it is 17.38%; for the "research governance" class, it is 13.36%; for the "commercialization" class, it is 18.06%; for the "biosciences" class, it is 22.17%; and for the "labeling" class, it is 20.16%.

In the factoring plane (Fig 2), we can see that the second opposition contrasts legal and biological objects (illustrated by discourses on “labeling” and “biosciences”) with the use of GM products in several social worlds (illustrated by discourses on “commercialization,” “research governance,” and “agricultural practices”).

In this graphic projection, the classes of words present in the left quadrants are marked by an increased institutional certification (whether political, economic, or legal governance), while those arranged on the right designate “raw” biological elements (biosciences or agriculture) without institutional scope. The classes of words in the upper quadrants are presented as legal or biological objects, while the lower quadrants more closely echo the use of these objects

(in the context of industrial or agricultural practices).

Four distinct lexical worlds emerge from these two lines of opposition: biology, industry and research governance, regulation, and agriculture.

The first dividing line is between the worlds of biology and genetic engineering techniques on the one hand and the practicing of research itself—whether private or public—and the economic debates to which it is linked, on the other. The world of biology does not mix with the market and research worlds. The former (to which 15% of the text segments are attributed) focuses on plants and their genes which are modified, edited, and cultivated: it describes the processes by which scientists create new biological traits. There is an extensive discussion of genetic engineering techniques, but no discussion of the end products: the lexical realm of science does not cover economic issues. It is mainly scientists (chi2 207.13, p < 0.0001) who contribute to this class of words, with the aim of popularizing science, via websites (chi2 28.42, p < 0.0001) or, less often, via blogs (chi2 13.71, p = 0.00021). However, although genetic engineering techniques are controversial, scientists rarely venture into this controversy, and address the general public (chi2 20.99, p < 0.0001) primarily on scientific issues (chi2 396.92, p < 0.0001) and mainly in a European context (chi2 6.96, p = 0.00833).

**Fig 3:**
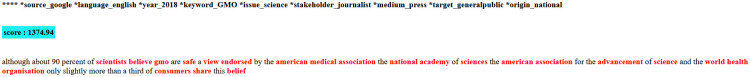
Example of a text segment using the word “science” in the English corpus.

Public debate arises when the scientific discussion moves toward economic issues and away from technical ones. Thus, "commercialization" (accounting for 14.7% of the text segments) is part of the language about science (class “research” accounting for 25.5% of the text segments) (Figs 1 and 2). The word “GMO” is strongly associated with the class “research” (chi2 84.03, p < 0.0001), as well as words such as “science” (chi2 303.3, p < 0.0001), “health” (chi2 218.51, p < 0.0001), “public” (chi2 156.34, p < 0.0001), “safe” (chi2 134.4, p < 0.0001), “consumer” (chi2 136.15, p < 0.0001), and “believe” (chi2 74.82, p < 0.0001). For example, as shown in Fig 3, “science” is used in institutional contexts.

This class tends to represent scientists’ individual views on issues of science governance, through the use of blogs (chi2 26.72, p < 0.0001).

**Fig 4:**
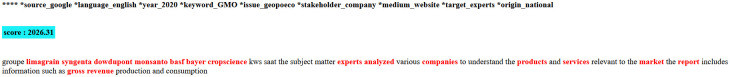
Example of a text segment using the word “Monsanto” in the English corpus.

For the “commercialization” class, words such as “Monsanto” (chi2 474.01, p < 0.0001), “company,” (chi2 419.89, p < 0.0001) “business” (chi2 145.68,, p < 0.0001), “corporation” (chi2 124.43, p < 0.0001), and “trade” (chi2 119.84, p < 0.00101) predominate. The products of genetic engineering have no other characteristic than to be exchanged on a market—like any other product, as we can see in the text segment from the word “Monsanto” (Fig 4).

For the “research governance” class, the main issue is also scientific (chi2 91.39, p < 0.0001), and in this case it is also scientists who communicate (chi2 33.13, p < 0.0001), directed at the general public (chi2 30.9, p < 0.0001), and generally in national contexts (chi2 14.75, p = 0.00012). The issues and actors are different for the "commercialization" class. The issues are primarily economic and geopolitical (chi2 604.04, p < 0.0001) as well as advertising (chi2 154.09, p < 0.0001), while it is citizens advertising (chi2 252.88, p < 0.0001), journalists (chi2 66.7, p < 0.0001), and farmers (chi2 59.59, p < 0.0001) who communicate toward experts (chi2 47.69, p < 0.0001), in an international (chi2 16.2, p < 0.00700), or non-specifiable (chi2 22.97, p < 0.0001) context.

Two other classes are decisive in the explanation of the corpus: the “labeling” class, accounting for 19.65% (1106/5628) of the classified text segments, and the “agriculture” class, accounting for 25.14% (1415/5628) of the classified text segments. They are both well dissociated from the other and isolated from classified text segments (Figs 1 and 2).

The second use of the term GMO or GMOs (chi2 113.62, p < 0.0001) can be found in the “regulation” class. The lexical realm of labeling describes institutionalization processes—mostly in Europe and the United States—with terms such as “label” (chi2 401.78, p < 0.0001), “eu” (chi2 368.89, p < 0.0001), “law” (chi2 74.17, p < 0.0001) “ban,” (chi2 184.13, p < 0.0001) “directive” (chi2 286.37, p < 0.0001) but also “bioengineered,” (chi2 167.37, p < 0.0001) “mutagenesis,” (chi2 73.74, p < 0.0001) and “approval” (chi2 76.67, p < 0.0001). The main issue of this class is to regulate (chi2 1420.3, p < 0.0001): to prohibit, to approve, to authorize genetic engineering techniques—using mutagenesis or not. The texts are mainly from organizations (such as regulatory bodies, non-profit organizations, political parties, associations) (chi2 163.34, p < 0.0001) who are communicating on the regulation and labeling of GMOs, targeting experts such as professionals in the agri-food industry (chi2 384.82, p < 0.0001). The origin of the texts is generally European (chi2 25.28, p < 0.0001) and national (chi2 17.8, p < 0.0001).

Finally, issues related to agriculture are evoked in the lexical realm of “agricultural practices”. This class includes words referring to crops, farmers, their work, the products used (“pesticide”, chi2 297.13, p < 0.0001), and the effects these products have on ecosystems (“insect”, chi2 256.33, p < 0.0001; “resistant”, chi2 200.72, p < 0.0001). Unsurprisingly, the main issues in this class are agriculture and biodiversity (chi2 345.24, p < 0.0001). These issues are found to be independent of each other and of the previous two. For the “regulation” class, even though newly regulated scientific techniques—such as CRISPR-Cas9—could be at the heart of the legal analysis, they are completely disconnected from it: “dna” is an anti-profile of the class (chi2–37.04, p < 0.0001). This is also the case for the agricultural and economic consequences of legal decisions: the issues “bioagri” (chi2–134.1, p < 0.0001), and “geopoeco” (chi 2–77.91, p < 0.0001) are antiprofiles. The legal issue seems to be fueled for itself and by itself, without taking into account other types of arguments, and without integrating the public debate—the texts are above all targeting experts.

### GMOs in the French-speaking world: A controversial term tied to risk issues

**Fig 5:**
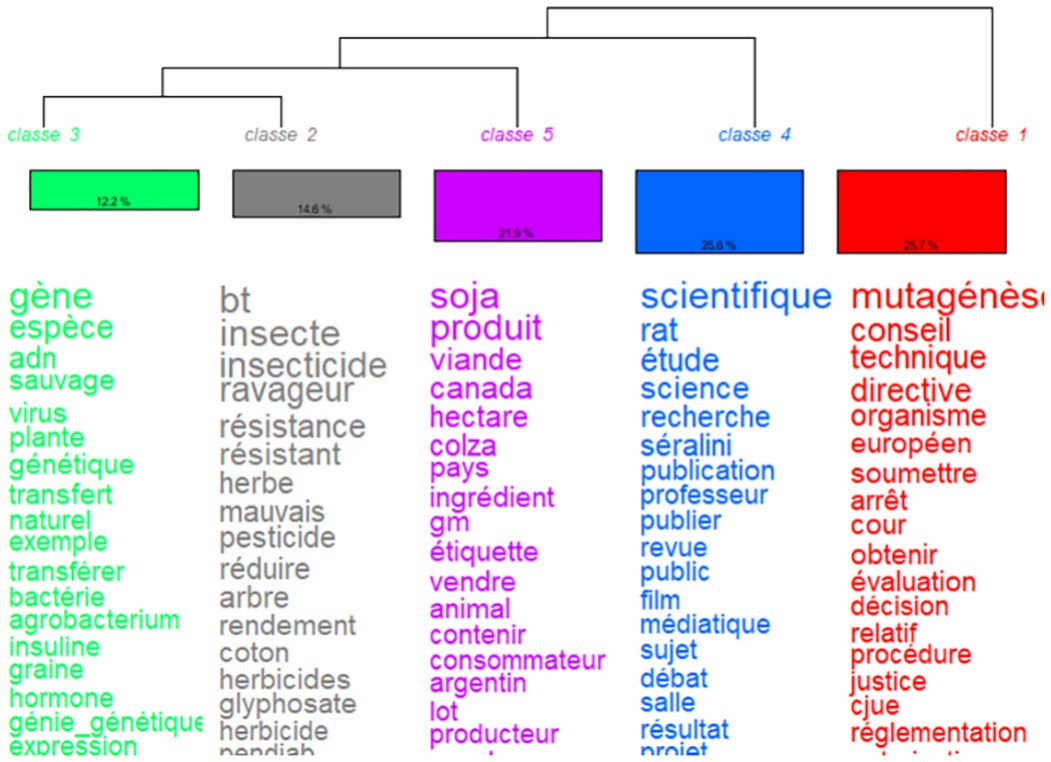
Descending Hierarchical Classification of the French corpus.

**Fig 6:**
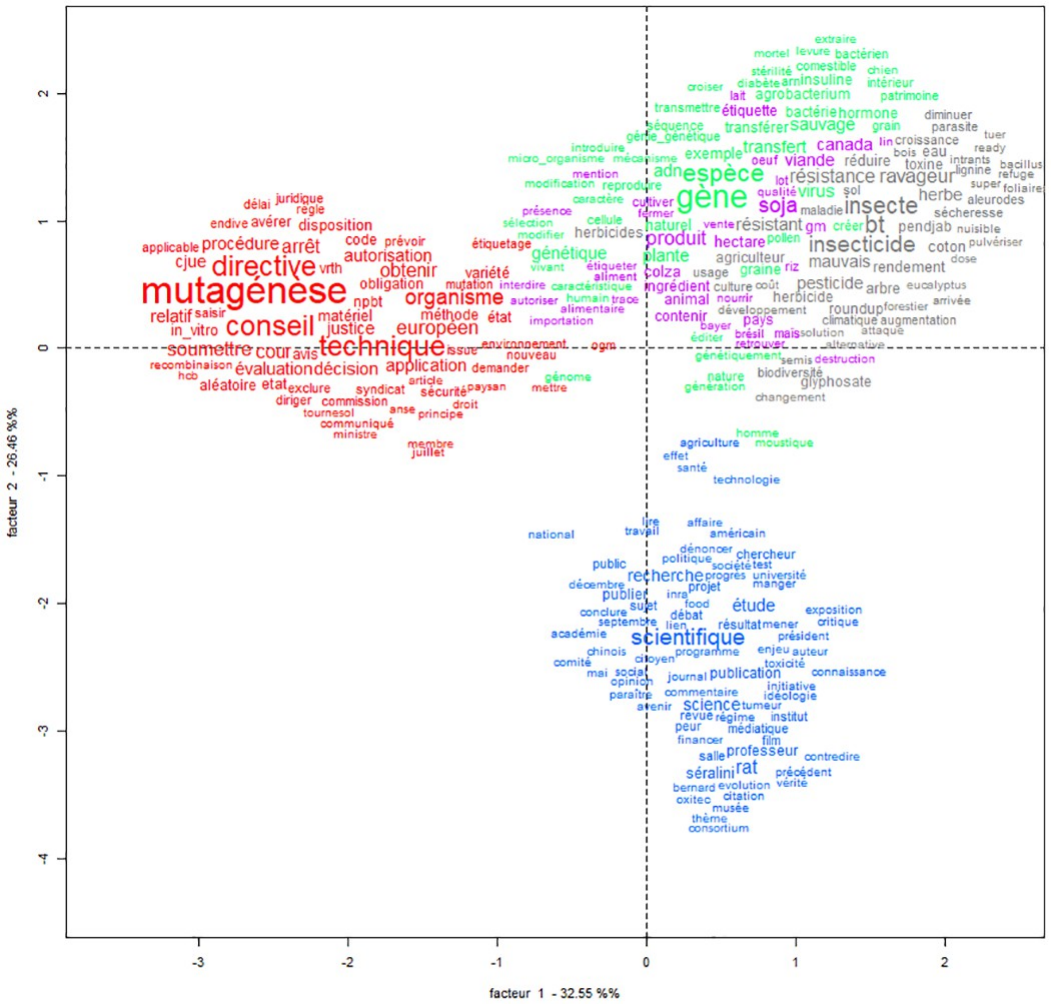
Correspondence factor analysis (CFA) of classes produced by Descending Hierarchical Classification (DCA) of the French corpus. Font size proportional to frequency by class. Horizontal axis, factor 1, 32.55%—Vertical axis, factor 2, 26.46%.

The French corpus is also structured around two major lines of opposition (see Figs 5 and 6, projection on the factorial plane of the word classification of the French corpus, Table 5). The first opposition is between “regulatory issues”, essentially European issues (in red), and socioeconomic issues, mainly considered as risk issues (all other colors). The second opposition is between “food products” (in pink), “agriculture” (in gray), “biology” (in green), and “science” (in blue). The total variance of the correspondence analysis is 98.46%. The observed variance for each class is 22,90% for "regulatory issues," 18,32% for “agriculture”, 18,56% for "biology”, 19,81% for “science” and 18,87% for “food products”. All the classes contribute significantly to the explanation of the total variance, which shows that they are highly relevant for the interpretation of the data structure.

In this graphic projection, the classes of words in the left quadrants essentially come from the legal world, while those on the right refer more to the rest of society (environment, economy, management of people and production of knowledge). The classes of words present in the upper quadrants mark risks and their management while the lower quadrants refer to the administration of things and knowledge.

**Table 5:** The 30 most frequent words in the French corpus.

Form	Freq.	POS
ogm	1454	nr
plante	398	nom
génétiquement_modifié	366	nr
organisme	363	nom
culture	341	nom
technique	330	nom
européen	254	adj
produit	246	nom
produire	245	ver
animal	230	adj
scientifique	229	nom
nouveau	224	adj
mettre	223	ver
variété	217	nom
mutagénèse	215	nr
utiliser	214	ver
agriculture	210	nom
maïs	209	nom
gène	209	nom
état	204	nom
génétique	197	adj
risque	192	nom
recherche	191	nom
santé	188	nom
environnement	183	nom
permettre	182	ver
france	180	nr
modifier	169	ver
obtenir	162	ver
conseil	161	nom

These two axes of opposition structure three lexical realms whose content is very different from those of the English-language corpus: regulation, environmental risks and issues, and the production of scientific knowledge.

As in the English-language corpus, regulatory issues are addressed in a separate forum. All other colors are opposed to this class: the issue is considered as independent from all the others (see Fig 5). These regulatory issues focus primarily on the legal controversy over new genome editing techniques. The term “mutagénèse” (‘mutagenesis’. chi2 528.43, p < 0.0001) figures prominently, with reference to the 2018 decision by the Court of Justice of the European Union to regulate these techniques in the same way as the earlier transgenesis techniques. But there are also terms that activists use to refer to NPBTs, such as “Varieties Made Tolerant to Herbicides/Variétés rendues tolérantes aux herbicides (VrTH)” (chi2 92.86, p < 0.0001) or “OGM cachés” (‘hidden GMOs’, chi2 37.73, p < 0.0001). This labeling enables them to be included in the EU regulatory framework on GMOs.

Debates on agriculture and environmental risks are highly visible and have a separate lexicon (class “agriculture” and class “biology”). But they only exist in the context of food production (class “food products”, Fig 5). Issues related to the “environmental impacts” of GMOs (class “agriculture” and class “biology”), where terms such as “insect” (“insect” chi2 256.25, p < 0.0001), “pesticide” (chi2 113.83, p < 0.0001), “roundup” (chi2 34.34, p < 0.0001), “species” (“espèce”. chi2 264.03, p < 0.0001), “gene” (“gene”. chi2 398.09, p < 0.0001), “wild” (“sauvage”. chi2 145.09, p < 0.0001), and “natural” (‘naturel’ chi2 100.04, p < 0.0001) appear, are interwoven with discussions about “food products” (in pink) with terms such as “product” (“produit”. chi2 214.09, p < 0.0001) and “soy” (“soja”. chi2 2014.69, p < 0.0001). This indicates the prominence of discourses on information provided to consumers about what they eat, as well as the risks to their health and the environment.

Unlike in the English-language corpus, environment and biology are tied to economic issues. This “food products” class is characterized by agriculture and biology (chi2 191.15; p < 0.0001), and also by advertising and marketing (chi2 174.4, p < 0.0001), which accounts for the fact that the stakeholders are mainly farmers (chi2 56.56, p < 0.0001) and companies (chi2 38.0, p < 0.0001), and to a lesser degree organizations (chi2 26.77, p < 0.0001). The origin is not French, but Canadian (chi2 168.81, p < 0.0001) or Swiss (chi2 27.2, p < 0.001).

The non-French origin is also relevant for the class “agriculture” and the class “biology”. For the “agriculture” class, the origin is international (chi2 35.1, p < 0.0001) and the main issue is science (chi2 33.97, p < 0.0001), followed by agriculture and biodiversity (chi2 10.26, p = 0.00135), and politics and economics (chi2 7.25, p = 0.0076). For the “biology” class, science is also the main issue (chi2 132.44, p < 0.0001). The texts target the general public (chi2 31.09, p < 0.0001) and have been written by citizens (chi2 24.77, p < 0.0001). The origin is generally international (chi2 16.34, p = 0.01130).

Finally, the scientific issues related to GMOs are strongly embedded in a French national context (chi2 15.34, p < 0.0001) and influence the European debate (chi2 4.13, p = 0.04212). In the lexical field of scientific production, there are terms such as “scientist,” (“*scientifique*”. chi2 235.02, *p* < 0.0001) “mediatic” (“*médiatique*”, chi2 47.86, *p* < 0.0001), and “fear” (“*peur*”. chi2 42.84, *p* < 0.0001). This class represents a large part of the text segments (25.64%) and, unlike the English corpus, is completely independent from the others (Figs 5 and 6), especially from the economic issues. The field of “scientific knowledge” refers to scientific organizations (“universities,” “institute,” “journal”), to the democratization of expert knowledge (with terms such as “debate,” “society,” “research,” etc.), or to risk management issues. It is not citizens, public organizations, or companies who are most vocal on these issues: rather it is scientists who, through the mainstream press (chi2 7.61, p < 0.0001) or blogs (chi2 159.9, p < 0.0001), are the most eager to convey these views (chi2 192.45, p < 0.0001), targeting the general public (chi2 22.82, p < 0.0001).

In addition, the very use of the term GMO is the subject of partisan discussions which include criticism of how GM products are engineered and regulated. GM products are controversial, as are the techniques used to produce them, and controversial researchers are cited, such as the French biologist Gilles-Eric Séralini who authored a contentious paper about the carcinogenic effects of GM food in rats (“rat”: chi2 160.42, *p* < 0.0001; “séralini”: chi2 105.58, p < 0.0001); or the Chinese researcher He Jiankui, who was the first to use CRISPR-Cas9 in humans and announced the birth of genome-edited twin girls in 2018. There is a more militant narrative about GMOs in French debates, directed against the scientific institutions that engineer these products.

## Discussion

Our research has produced two main sets of findings. The first is that the French- and English-language corpora shared some characteristics. First, the issues related to the regulation of GMOs are framed by and for experts, without involving public debate or other types of actors. The rest of the issues are addressed without mentioning the regulatory elements for GMOs. The legal realm has not entered into public debate, even though the period chosen for the scope of this study was just after the CJUE’s 2018 decision to include NPBTs within the EU legislation on GMOs. As these are essentially organizations with an institutional discourse related to regulation (mainly, regulatory bodies, non-profit organizations, political parties, and associations), we hypothesize that this semantic proximity between the English and the French corpora, is partly due to translation from one language to the other. Indeed, French organizations of this kind are likely to have translations of their web pages in English, and/or to use content produced in English by European institutions for their French websites. Secondly, scientific controversies are absent when English is used as a global language (in particular, by international organizations and corporations). They are present only in the French-language corpus and in the part of the English-language corpus that deals with national issues. This is a major difference between the globalized vs. localized framing of the information circulating about GMOs on the Internet.

The second set of findings has to do with significant differences between the French and English corpora. The French-language corpus is characterized by the strong association of genetic engineering with matters relating to risks, particularly environmental ones. This is consistent with studies that found risk to be an important frame [16]. Of particular interest here is that controversies focus on the way in which genetically engineered products are made—by attacking in particular the institutions of expertise. This resonates with the long institutional history of GMO regulation in France. In France, risk issues remain a privileged terrain for complex professional regulation under the umbrella of the state, while in the United States there has been an increasing reliance on industrial regulation [32]. In addition, consumer, farmer, and environmental interest groups have proclaimed second-party expertise and garnered support with the French public for the non-implementation of the EU regulation on agricultural biotechnology [33]. The class referring to this type of negative framing of GMOs and NPBTs appears alone, and is disconnected from the others. Indeed, we were surprised not to find a lexicon directly associated with genetic engineering techniques and the way GMOs are produced. The term “GM” is frequently used in these controversies but not precisely defined: it is used as a generic label (whether to characterize GM products or to discuss their environmental impacts) or even as a catchword in support of the claims of citizen science [34]. It is a part of critical discourse, which challenges public institutions and pressures them to regulate these “new selection techniques.”

It stands in contrast with the globalized English-language communication, used by large companies and by the European Union to communicate with European citizens, especially on scientific issues. This communication emphasizes more descriptive, more consensual, and less militant issues. Indeed, the English-language corpus focuses on the technical dimensions of genetic engineering and on the end products: how to regulate, cultivate, and integrate them into the market and into consumer practices. It tends to frame GMOs as products to be traded on a market.

Communication on GMOs is not linked to concerns about the way in which science relates to society and does not refer to citizens. The global English-language political culture is not very critical of GMOs; the lexicon used on the web is not controversial. The economic dimension is very present, the description of existing regulations is central, while the problematization in terms of risks is almost non-existent.

Our research has several implications for research and practice. The first is that it calls for expanding the field of studies on the media framing of GMOs—and more broadly, of controversial scientific topics—by taking into account digital media and their cross-border nature. So far, most studies of the media framing of biotechnologies are based on survey data and proceed by country, for example France, United States, Hungary, United Kingdom [3,18,35], focusing on the opinion-leading press. Comparisons tend to be made between countries. National political cultures have had an impact on the regulation of GMOs, whether in terms of the principles that inform regulatory decision-making, the perception of risks around organisms referred to as “genetically modified” (GM), or trust in biotechnology and consumers’ attitudes toward GM food [36], and cultural values regarding genetic modification or gene editing [37] may have a decisive impact on food consumption. Yet, as our study shows, digital media play a key role in making apparent the place of biotechnology in the social, legal, and economic fields. Therefore, access to different types of information circulating on the Internet, and the prevalence of certain framings of debates according to language, are important subjects of investigation for understanding how media framing operates online. Among the questions that are beyond the scope of our research, but which deserve to be investigated, is the question of social differences in access to online information. Just as it is not the same social categories that read tabloids, watch cartoons, or read the “elite press”, we assume that it is not the same categories that access information in a globalized public sphere as opposed to a national public sphere on the internet.

A second implication of our findings is that they call for further research on the consequences of these different framings of GMOs-related debates on the Internet on public attitudes towards GMOs. Our findings for the French-language corpus resonate with trends observed in a recent opinion survey that shows increased awareness of environmental risks and increased biological conservatism about French respondents [38]. Our research also echoes European and national barometers that regularly show a more pronounced mistrust in France than in other European countries with regard to genetic modifications used for various purposes (medicine, agriculture, world hunger) [39]. Among biotechnologies, genome editing tools are the subject of more marked mistrust.

While our article does not aim to test the effect of the online framing of genetic engineering issues on public attitudes, it is reasonable to assume that there is a relationship between these, as suggested by a study conducted by Wang at al. [13] that shows that representations of genetically modified organisms in cartoons on the internet in China are associated with strong political polarization. Understanding the impact of communications online on public attitudes is important for policy-makers, who also take into account public perception of risk in regulatory decisions [40]. Adding to internet sites, the echo chamber of social media—with potential aggregation in homophilic clusters of users that dominate online [24]—probably intervenes in public attitudes to biotechnologies, and also deserves scholarly attention.

Lastly, our study has implication for public and institutional communication on GMOs. It is beyond the scope of this research to make recommendations for specific situations.

However, we argue that institutional communication on scientific issues cannot ignore how these issues are received by citizens. This seems especially important for international institutions, such as the European ones, to recognize that how communication about GMOs is received by the public depends on the language used. In the French lexical field, genetic engineering tools raise not only technical issues, but also democratic questions. Moreover, the use of the label “GM” is associated with militant postures, which is not at all the case in the global English-speaking culture. Taking these cultural differences into account while communicating could help the public understand cutting-edge scientific issues. In particular, misunderstandings could be tempered using appropriate terminology around genetic engineering products and tools. Notably, our study suggests that the label “GM” should only be used with caution, and could even be avoided, as this generic term is used as a shorthand to simplify a complex issue and facilitates cultural bias in the reception of communication.

## Conclusion

In this article, we use content that is publicly available on the internet as a prime site for observing how the significations of what is “genetically modified” are framed in a specific language (French) vs a global language (English), with the aim of drawing lessons for public and institutional communication in biotechnology. We found significant heterogeneity in approaches to biotechnology, including regulatory decision-making and public attitudes toward biotechnologies, as well as political discourses and institutions that relate GMOs more broadly to political, social, and economic activities and values. The media play an important role in framing the attention given to biotechnologies and in the sociocultural perception of risks. The governance issues surrounding GMOs and their potential for generating public controversies require us to take into account the relationship between scientific culture, sources of information and the public’s overall perception of genetic engineering.

## Supporting information

S1 File (XLSX)

S2 File (XLSX)
